# Stachydrine ameliorates hypoxia reoxygenation injury of cardiomyocyte via enhancing SIRT1-Nrf2 pathway

**DOI:** 10.1186/s13019-023-02363-6

**Published:** 2023-09-26

**Authors:** Xi Zhu, Yingbiao Wu, Xiaogang Zhang, Wei Gu, Zhongping Ning

**Affiliations:** Department of Cardiology, Shanghai University of Medicine & Health Sciences affiliated Zhoupu Hospital, No.1500 Zhouyuan Road, Pudong New District, Shanghai, 201318 China

**Keywords:** Hypoxia/reoxygenation (H/R) injury, Stachydrine, Cardiomyocyte, SIRT1, Nrf2

## Abstract

**Background:**

Hypoxia/reoxygenation (H/R)-induced cardiomyocyte cell apoptosis is critical in developing myocardial infarction. Stachydrine (STA), an active constituent of Leonurus heterophyllus sweet, could have a protective effect on myocardial H/R injury, which remains unexplored. Therefore, the study aimed to investigate the protective effects and mechanisms of STA on H/R injury of cardiomyocytes.

**Methods:**

Rat cardiomyocyte H9c2 cells underwent H/R (hypoxia for 4 h and reoxygenation for 12 h). Cells were pretreated with STA (50 µM) 2 h before H/R. Cardiomyocyte injury was evaluated by CCK-8 assay and lactate dehydrogenase (LDH) release. Apoptosis was assessed by TUNEL staining and caspase-3 activity. Oxidative stress was assessed by lipid oxidation product MDA and a ROS-scavenging enzyme SOD in culture media. Western blot was performed to measure the protein expressions of SIRT1, Nrf2, and heme oxygenase-1 (HO-1).

**Results:**

STA reversed the decrease in cell viability and increased LDH release in H9c2 cells with the H/R insult. STA significantly suppressed oxidative stress, reduced MDA content, and increased SOD activity in H9c2 cells exposed to H/R. STA reduced apoptosis in H9c2 cells exposed to H/R, as evidenced by the reduced TUNEL positive cells and caspase-3 activity. In addition, STA enhanced SIRT1, Nrf2, and HO-1 protein expression in H/R-stimulated H9c2 cells. SIRT1 and Nrf2 involved the protective effect of STA in H/R-exposed H9c2 cells, as the changes in cell viability and caspase-3 activity by STA can be reversed by SIRT1 inhibitor EX-527 or Nrf2 siRNA.

**Conclusions:**

Our data speculated that STA protects H/R injury and inhibits oxidative stress and apoptosis in cardiomyocytes by activation of the SIRT1-Nrf2 pathway.

**Supplementary Information:**

The online version contains supplementary material available at 10.1186/s13019-023-02363-6.

## Introduction

Myocardial ischemia, also called cardiac ischemia or hypoxia/reoxygenation (H/R)-induced heart damage, is caused by decreased blood flow [1]. Atherosclerosis, coronary artery spasm, and blood clots are the typical causes of H/R [2]. Heart attack, heart failure, and arrhythmia may occur with the progression of H/R, increasing the fatality rate [3, 4]. In severe cases, 15% of H/R patients will die before hospitalization, and 15% will die while receiving medical care. Even worse, about 10% of patients will die within a year of being discharged [5, 6]. Angioplasty or bypass operations are typically used to address H/R [7], whereas injuries are generally irreversible. Therefore, exploring new therapeutic agents and investigating their potential mechanisms underlying myocardial H/R injury is important.

Stachydrine (STA) is an active component of Leonurus heterophyllus sweet, which is also named “Yi Mu Cao” or “mother-benefiting herb” in traditional Chinese medicine. Leonurus heterophyllus sweet has demonstrated pharmacological effects on ischemic diseases in experimental and clinical studies, with improved coronary blood flow, platelet aggregation, and improved heart function [8]. In addition, recent studies showed that components extracted from Leonurus heterophyllus sweet alleviated left ventricular dysfunction or remodeling in animal models. Furthermore, STA prevented cardiomyocyte hypertrophy induced by norepinephrine in vitro study [9]. However, the effects and the detailed mechanisms of STA in myocardial H/R injury are unknown.

Silent information regulator transcript-1 (Sirt1) is a histone deacetylase that is nicotinamide adenine dinucleotide (NAD+) reliant and closely associated with several cellular processes, including cell metabolism, aging, apoptosis, inflammation, and oxidative stress [10]. Sirt1 has the ability to control crucial transcription factors like nucleus erythroid factor 2-related factor 2 (Nrf2), which is crucial for cytoprotection, the anti-inflammatory response, and the antioxidant response [11]. In the leucine zipper transcription factor family [12], Nrf2 is an antioxidant sensor and regulator of intracellular ROS preparation. The expression of the antioxidant protein is stimulated to perform an antioxidant function after Nrf2 has been triggered, whereas the amounts of ROS are restrained [13]. However, the role of STA on the H/R injury of cardiomyocytes through the SIRT1-Nrf2 pathway remains unexplored.

In this study, we hypothesized that STA could attenuate myocardial H/R injury. To investigate this hypothesis, the H9c2 cardiomyocyte cell line was used to establish an in vitro myocardial H/R injury model to explore the roles and potential mechanisms of STA in myocardial H/R injury. The results showed that by activating the SIRT1-Nrf2 pathway, STA protects against H/R injury and prevents oxidative stress and apoptosis in cardiomyocytes.

## Methods

### Cell culture

The cardiac myoblast cell line H9c2 was purchased from the Cell Bank of the Chinese Academy of Sciences (Shanghai, China) and cultured in high‑glucose DMEM (Gibco; Thermo Fisher Scientific, USA) supplemented with 10% fetal bovine serum (FBS) in a 5% CO2 incubator at 37˚C. After growing to 70‑80% confluence, cells were undergoing serum starvation in DMEM with 0.1% FBS for 24 h.

### H/R injury model

H/R injury was made in H9c2 cells by exposing them to a hypoxic atmosphere (1% O_2_) for 4 h, followed by reoxygenation for 2 to 16 h. The control cells were kept under normoxic conditions. In addition, H9c2 cells were pretreated with STA (Cat no. HY-N0298, MedChemExpress, USA) for 2 h before H/R.

### Experimental protocols

To determine the optimal conditions of H/R injury, H9c2 cells were divided into seven groups: control group was cultured in normoxic conditions; hypoxia group was exposed to the hypoxic atmosphere for 4 h; H/R group were exposed to the hypoxic atmosphere for 4 h, and then underwent reoxygenation for 2, 4, 8, 12 or 16 h. To determine the optimal concentration of STA, cells were incubated with 10, 20, 50, 100, and 200 µM of STA for 18 h under normoxic conditions.

To investigate whether STA inhibits H/R injury and explore the related mechanisms, cells were divided into four groups:

1. Control group (normoxic conditions).
2. STA group were preincubated with 50 µM of STA and cultured under normoxic conditions.
3. H/R group were exposed to the hypoxic atmosphere for 4 h, followed by reoxygenation for a further 12 h.
4. H/R + STA group were preincubated with 50 µM of STA for 2 h, followed by 4 h hypoxic atmosphere and 12 h reoxygenation.

To investigate whether SIRT1 or Nrf2 involve the effects of STA, cardiomyocytes were preincubated with 1 µM SIRT1 inhibitor (EX-527, Cat no. HY-15,452, MedChemExpress, USA) or transfected with Nrf2 siRNA for 24 h, followed by H/R injury. Next, cells were preincubated with 50 µM of STA and/or EX-527 for 2 h, followed by 16 h of H/R. Finally, cells were transfected with Nrf2 siRNA using Lipofectamine 2000 (final concentration: 80 nM) 24 h before hypoxia, and then cells were preincubated with 2 h of 50 µM STA 16 h of H/R. The control group was solely exposed to H/R.

### Cell transfection with Nrf2 siRNA

The H9c2 cells were plated in 6-well plates (2 × 10^5^ cells/mL), incubated until approximately 70% confluence, and then transfected with Nrf2 siRNA or negative control siRNA (synthesized by GenePharma, Shanghai, China) using lipofectamine 2000 reagent. Next, the cells were added with a 1000 µL transfection complex solution (contains 0.5 µg of Nrf2 or control siRNA constructs) at 37 °C for 8 h using lipofectamine 2000 reagent. After 6 h of transfection, the cells were incubated in a complete medium, and transfection efficiency was determined using western blot after 24 h.

### Cell viability assay

H9c2 cells were seeded in a 96-well plate (2 × 10^3^ cells in 100 µL media per well). After 18 h of various treatments, cells were added with CCK-8 solution (10 µL) in each well, followed by incubation at 37˚C for 2 h. The absorbance at 450 nm was measured using a microplate reader (MD, SpectreMax 190).

### LDH release assay

Cell injury was evaluated by measuring the released LDH in the supernatant of damaged cardiomyocytes. After 18 h of various treatments, the culture medium was collected to measure the amount of LDH using an LDH assay kit (Cat no. A020-2-2, Jiancheng, Nanjing, China). Cellular LDH amount was expressed as U/dL.

### Determination of MDA and SOD

H9c2 cells were harvested, and protein was extracted and quantified by the BCA assay kit (Beyotime Biotechnology, China). MDA (Cat no. S0131S) content was measured at 532 nm absorbance by a microplate reader and expressed as mmol/mg protein. SOD (Cat no. S0109) was measured at 520 nm absorbance and expressed as U/mg protein.

### Caspase-3 activity assay

Cells were lysed and then centrifuged at 1000 g for 10 min. The supernatant (30 µL) was co‑incubated with 90 µl caspase-3 substrate AC‑DEVD‑pNA (final concentration 0.2 mM, Sigma, St. Louis, MO, USA) at 37˚C for 2 h. The caspase-3 activity was evaluated by measuring absorbance at 405 nm using a microplate reader and then was normalized to the control group.

### Western blot

Protein was extracted from H9c2 cells using RIPA lysis buffer and quantified using a BCA assay kit. Protein (50 µg) was subjected to 10% SDS-PAGE electrophoresis and then transferred onto PVDF membranes. Membranes were then incubated with an antibody against SIRT1 (1:200, Abcam, UK), Nrf2 (1:100, Abcam, UK), HO-1 (1:100, Abcam, UK) and β-actin (1:1000) overnight at 4 °C. After washing with TBST, the membranes were incubated with HRP-conjugated secondary antibody (goat anti-rabbit IgG, 1:2000). The proteins were detected using ECL (Pierce Biotechnology, USA).

### Statistical analysis

Results were presented as mean ± standard deviation, and analyses were carried out by SPSS 19.0 software (SPSS, Inc., Chicago, IL, USA). Normality test was performed, and one-way ANOVA was used to compare differences of multiple groups, followed by the LSD method for further comparison between the two groups. P < 0.05 was considered as the criteria of statistically significant difference.

## Results

### STA alleviated H/R-induced cardiomyocyte injurie and oxidative stress

**Fig. 1:**
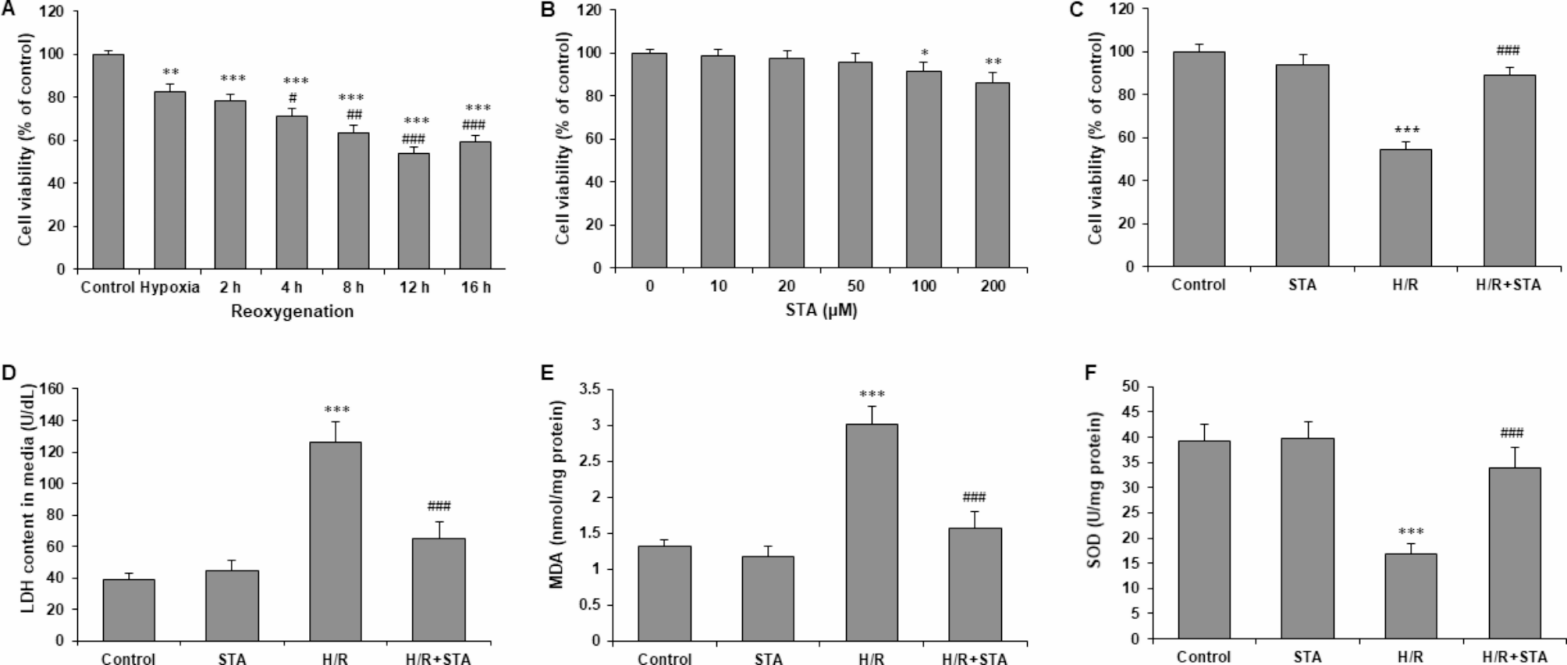
STA alleviates H/R‑induced cytotoxicity and oxidative stress in cardiomyocytes. **(a)** H9c2 cells were subjected to 4 h hypoxia and reoxygenation for 2, 4, 8, 12, or 16 h. **(b)** H9c2 cells were incubated with STA (0, 10, 20, 50, 100, 200 µM) for 24 h. **(c)** H9c2 cells were pretreated with 50 µM STA for 2 h, followed by 4 h hypoxia, and 12 h reoxygenation. Cell viability was evaluated by CCK-8 assay. **(d)** An LDH release assay evaluated cardiomyocyte damage. The colorimetric method determined the Lipid oxidation product MDA **(e)** and a ROS-scavenging enzyme SOD **(f)**. Values are expressed as Mean ± SD from at least three independent experiments. *P < 0.05, **P < 0.01, ***P < 0.001 vs. control group; #P < 0.05, ##P < 0.01, ###P < 0.001 vs. H/R group. STA, Stachydrine; H/R, hypoxia/reoxygenation; LDH, lactate dehydrogenase

H9c2 cells were subjected to hypoxia for 4 h, followed by reoxygenation for further 2, 4, 8, 12, or 16 h. Reoxygenation for 4, 8, 12, and 16 h markedly reduced cell viability compared with cells with hypoxia alone (Fig. 1(a)). Therefore, 12 h was selected as the optimal condition of reoxygenation for further experiments. To select the optimal concentration of STA, we then examined the effect of STA on H9c2 cells, and cells were pretreated with STA at 0, 10, 20, 50, 100, and 200 µM for 18 h. STA treatment showed no changes in cell viability at 10, 20, and 50 µM and significantly decreased cell viability only at 100 and 200 µM (Fig. 1(b)). We then selected 50 µM STA for subsequent experiments. H9c2 cells were pretreated with 50 µM STA for 2 h, followed by 4 h hypoxia and 12 h reoxygenation. STA significantly attenuated the decrease in cell viability in H9c2 cells with H/R alone (Fig. 1(c)). In addition, STA also significantly reversed H/R induced LDH release from H9c2 cells after H/R exposure (Fig. 1(d)). To investigate the effect of STA on oxidative stress, we measured intracellular oxidative stress indicators. H/R significantly increased MDA content and decreased SOD activity, which was reversed by STA pretreatment (both P < 0.001) (Fig. 1(e), 1(f)). These findings indicated that STA alleviates oxidative stress in H9c2 cells after H/R.

### STA ameliorated H/R-induced cardiomyocytes apoptosis

**Fig. 2:**
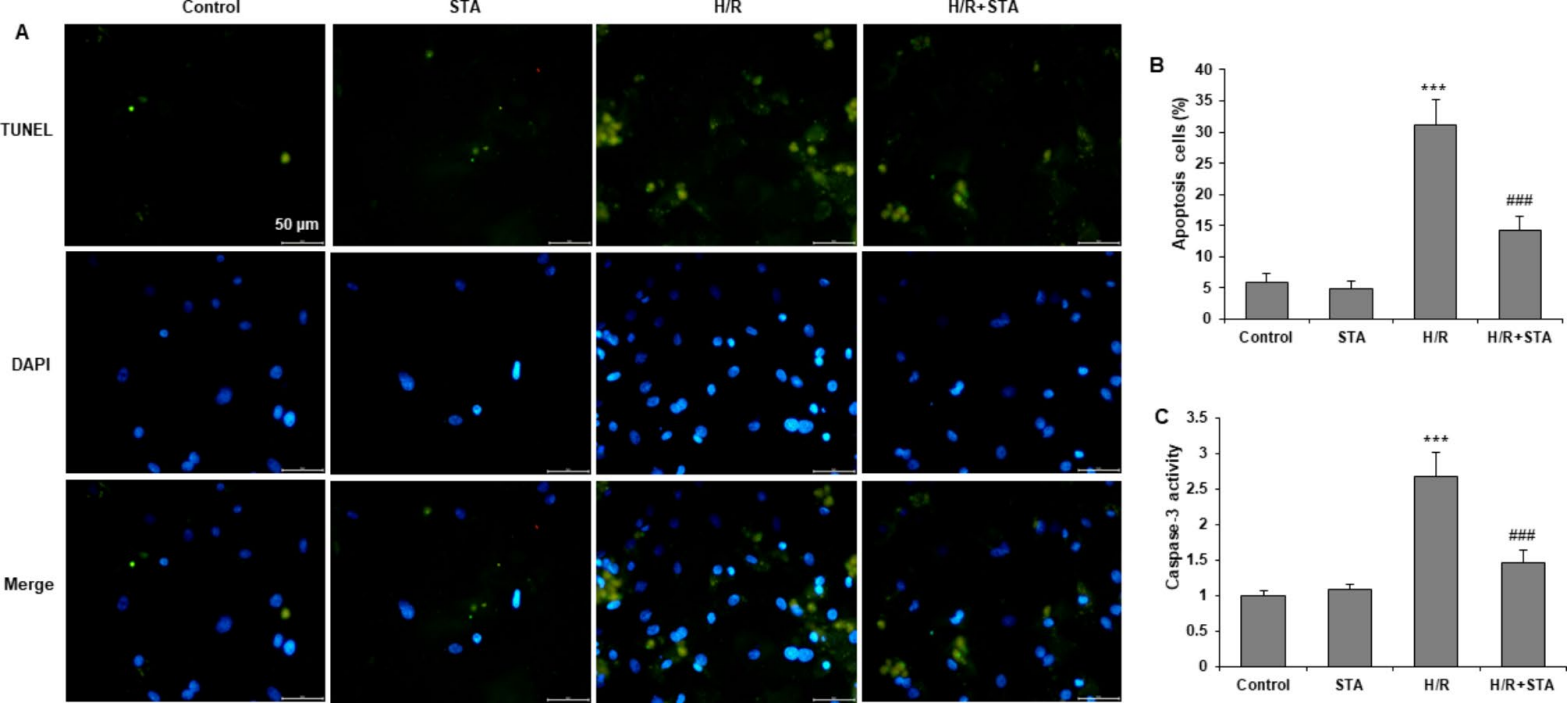
STA inhibits apoptosis in cardiomyocytes with H/R. H9c2 cells were pretreated with 50 µM STA for 2 h and then subjected to 16 h H/R. **(a)** Apoptotic cells were stained with TUNEL. Representative photomicrographs of H9c2 cardiomyocytes are shown. Magnification, ×50. **(b)** Quantification of TUNEL staining results. Apoptosis cells were counted from five random fields and normalized to total cells. **(c)** The caspase-3 activity was measured. Bar: 50 µM. Values are expressed as Mean ± SD from at least three independent experiments. ***P < 0.001 vs. control group; ###P < 0.001 vs. H/R group

H/R insult markedly increased the number of TUNEL-positive cells compared with the control group, which was significantly reversed by STA (Fig. 2(a)). Moreover, STA significantly attenuated the H/R induced decrease in apoptotic effect (Fig. 2(b)) and increased caspase-3 activity (Fig. 2(c)), with no apparent influence on H9c2 cardiomyocytes by STA alone. Thus, the results indicated that STA has an anti-apoptotic effect on H/R-induced cardiomyocytes.

### STA modulated SIRT1 and Nrf2/HO-1 anti-oxidative pathway

**Fig. 3:**
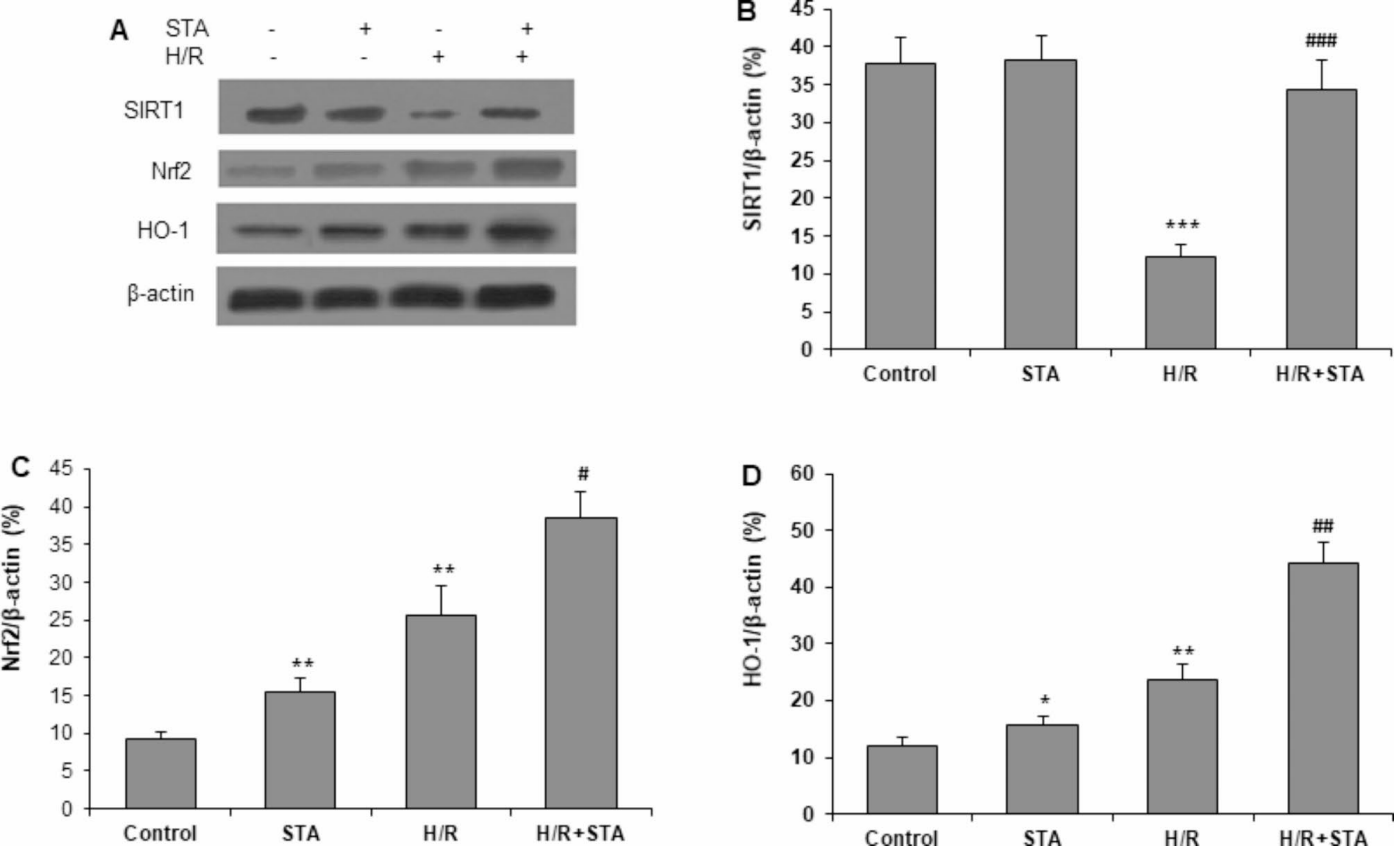
STA enhances expressions of antioxidant-related proteins in cardiomyocytes with H/R. H9c2 cells were pretreated with 50 µM STA for 2 h and then subjected to H/R for 16 h. **(a)** Western blots were performed, and the representative protein bands are shown. The mean relative levels of SIRT1 **(b)**, Nrf2 **(c)**, and HO-1 **(d)** in H9c2 cardiomyocytes are shown. Values are expressed as Mean ± SD from at least three independent experiments. *P < 0.05, **P < 0.01, ***P < 0.001 vs. control group; #P < 0.05, ##P < 0.01, ###P < 0.001 vs. H/R group

To explore the molecular mechanism underlying protective effects by STA, antioxidative protein levels were determined by western blot (Fig. 3a). H/R insult stimulated a decrease in mRNA and protein expression of SIRT1 and Nrf2, but these changes were reversed by STA pretreatment (Fig. 3(a), 3(b), 3(c)). We then measured Nrf2 and HO-1 proteins. H/R insult enhanced the expression of Nrf2 and HO-1 proteins in H9c2 cells. Pretreatment with STA further potentially increased the protein expressions of Nrf2 and HO-1 after exposure to hypoxia/reoxygenation (P < 0.01and P < 0.05) (Fig. 3(c), 3(d)). These results indicate that STA activates SIRT1 and Nrf2-HO-1 pathways, which might contribute to the protective effect of STA on H/R‑induced cytotoxicity and oxidative stress of H9c2 cells.

### SIRT1 and Nrf2 mediated the effects of STA in H/R-induced cells

**Fig. 4:**
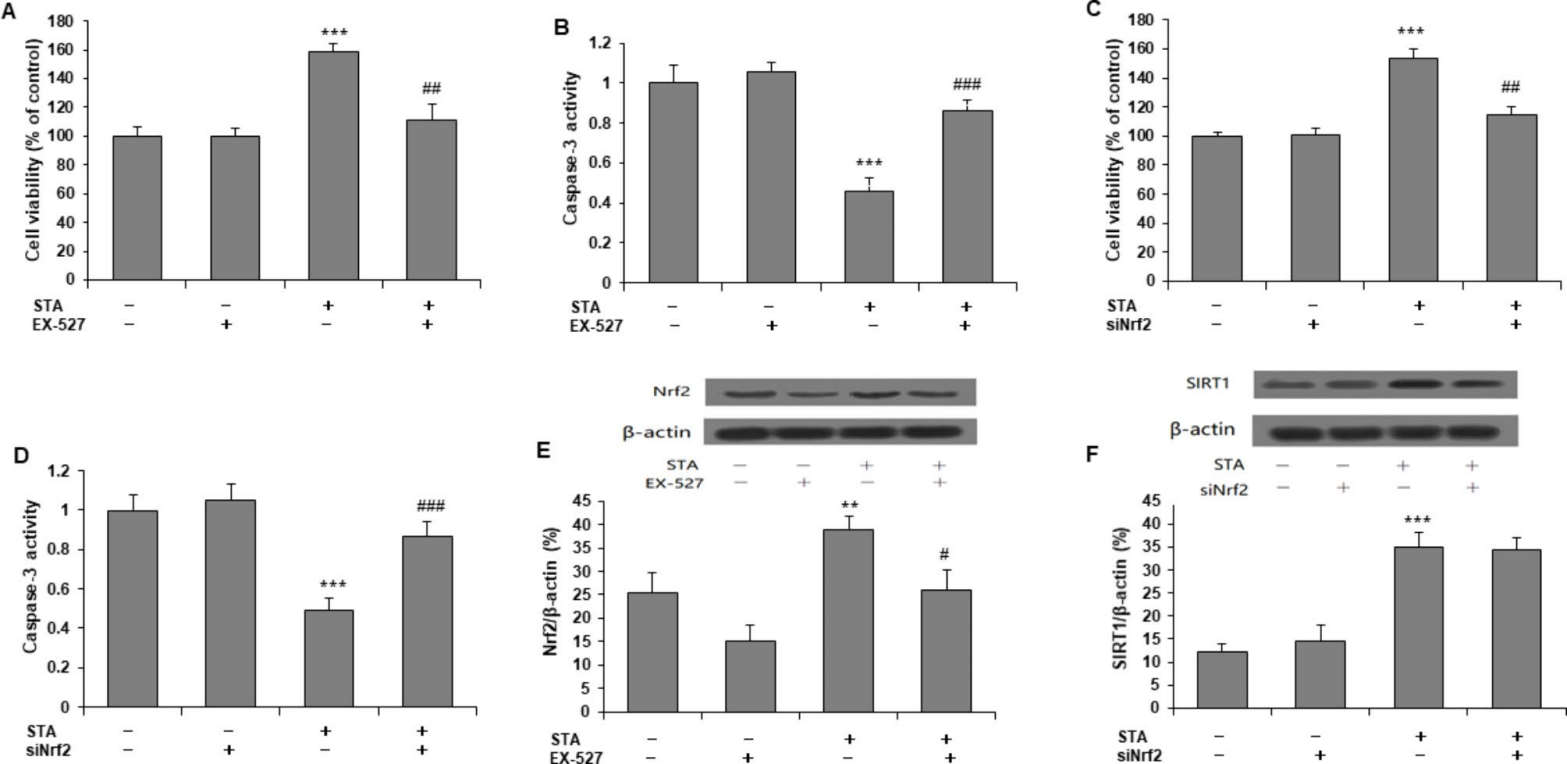
SIRT1 and Nrf2 mediate the protective effects of STA on H/R exposure-induced injury in H9C2 cells. H9c2 cells were exposed to 1 µM SIRT1 inhibitor (EX-527) or transfected with Nrf2 siRNA for 24 h, followed by incubation with STA (50 µM) and H/R for 18 h. EX-527 reversed the increase in cell viability **(a)** and decreased caspase-3 **(b)** by STA. Nrf2 siRNA also reversed the increase in cell viability **(c)**, and decrease in caspase-3 **(d)** by STA. Western blotting was performed to determine protein levels of SIRT1 and Nrf2, and representative immunoblots in H9c2 cells are shown. **(e)** EX-527 decreased nrf2 protein in a cell with H/R exposure. **(f)** The increase in SIRT1 protein by STA remains unchanged by Nrf2 siRNA. Values are expressed as Mean ± SD from at least three independent experiments. *P < 0.05, **P < 0.01, ***P < 0.001 vs. control group; #P < 0.05, ##P < 0.01, ###P < 0.001 vs. STA alone group

**Fig. 5:**
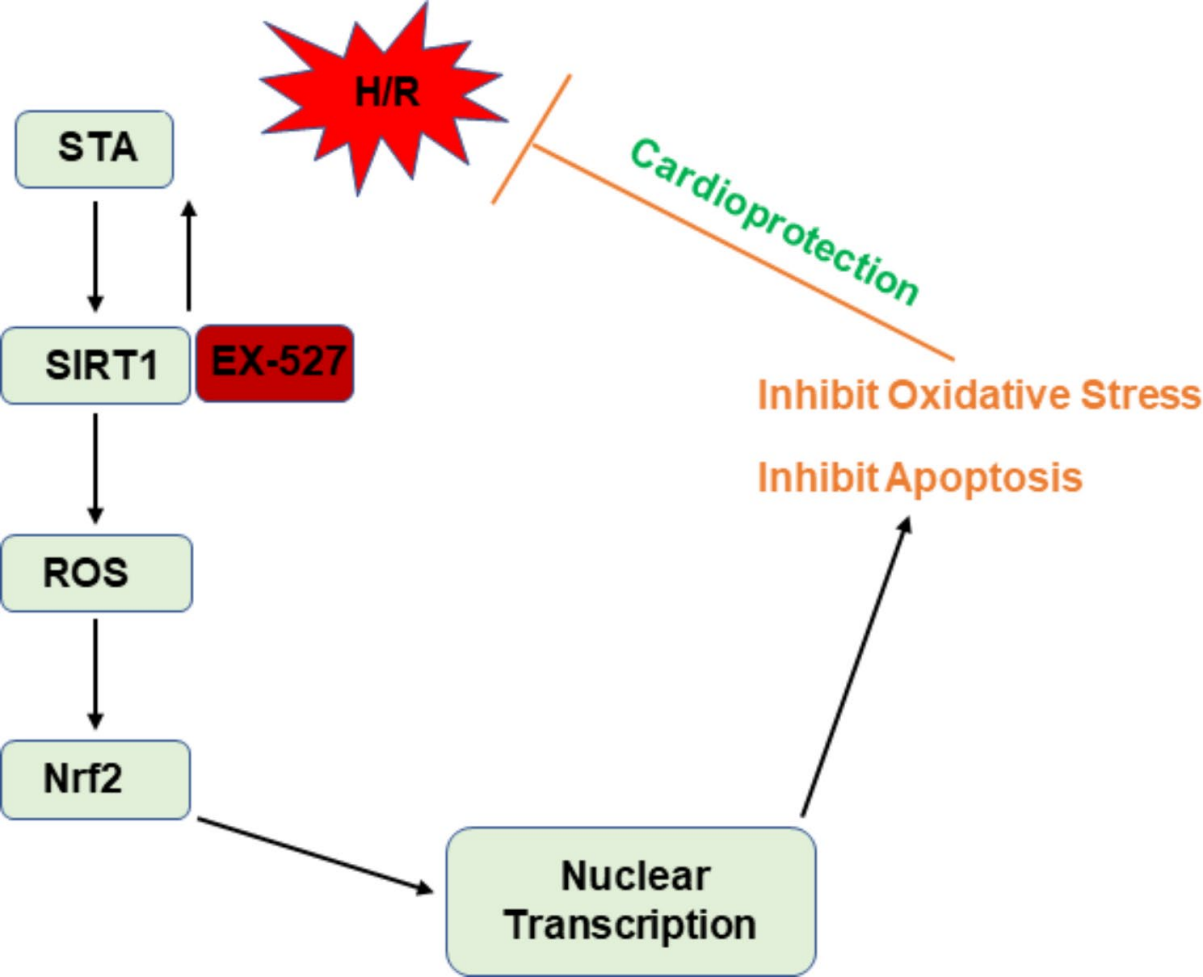
Schematic representation of the SIRT1-Nrf2 pathway regulated by STA.

To investigate the role of SIRT1 and Nrf2 in a protective effect on H/R injury of cardiomyocytes cells by STA, H9c2 cells were pretreated with SIRT1 inhibitor (EX-527: 1 µM), or transfected with Nrf2 siRNA for 24 h to inhibit the expression of SIRT1 and Nrf2, respectively. EX-527 reversed the increase in cell viability (Fig. 4(a)) and decreased caspase-3 (Fig. 4(b)) by STA. Knocking down Nrf2 by siRNA also reversed the increase in cell viability (Fig. 4(c), 4(d)) and decrease in caspase-3 (Fig. 4(d)) by STA. We then investigated the relationship between SIRT1 and Nrf2. Compared with H9c2 cells with H/R and STA, cells with EX-527 pretreatment significantly reduced protein expression of Nrf2 (Fig. 4(e)). However, Nrf2 siRNA transfection did not change the protein expression of SIRT1 in H9c2 cells with H/R and STA (Fig. 4(f)). These results indicated that SIRT1 and Nrf2 mediate the protection of H/R damage to cardiomyocytes by STA (Fig. 5), and SIRT1 may lie upstream of the Nrf2 pathway.

## Discussion

This study explored the effect and potential mechanisms of STA on myocardial H/R injury in H9c2 cells. Myocardial H/R significantly reduced the cell viability, increased LDH release, oxidative stress, and apoptosis in H9c2 cells, which could be reversed by STA pretreatment. The result implies that STA has the potential protective effect on myocardial H/R injury. Furthermore, the related mechanisms may be associated with enhanced expression of SIRT1 and Nrf2 proteins by STA in H9c2 cells with H/R insult.

Various biological changes are underlying H/R-induced myocardial injuries, such as ROS-induced oxidative stress, endothelial dysfunction, activation of apoptosis, and autophagy [14]. Both hypoxia and reoxygenation induce cardiomyocyte apoptosis by destroying microenvironment homeostasis [15]. Furthermore, oxidative stress is a significant inducer of cardiomyocyte apoptosis, which further aggravates myocardial H/R injury [16, 17]. This study demonstrated that H/R insult decreased cell viability, increased apoptotic rate and caspase-3 activity, increased MDA content, and decreased SOD activity in H9c2 cells. However, these effects were reversed by STA. The suppression of oxidative stress was also observed in other reports that STA inhibited oxidative stress in rats of carbon tetrachloride-induced hepatic fibrosis and isoproterenol-induced cardiac hypertrophy [18, 19]. Though STA has shown a suppressive effect on cardiomyocyte hypertrophy [9, 19, 20], this study firstly reported the role of STA in cardiac H/R-induced injury. Currently, the study on H/R injury by STA was only reported in a cerebral ischemia-reperfusion mice model [21]. STA also showed in vivo pharmacologic effects on cardiac fibrosis, bone loss and intervertebral disc degeneration [22–24], which suggests that it has potential function against cardiac H/R-induced injury in animal model [25]. Furthermore, pharmacokinetic study showed that STA has rapid absorption and excretion after oral administration in normal rats [26]. Given that leonine protected hypoxic neonatal rat cardiomyocytes and infarcted rat heart [27], it is reasonable that STA, the main active component of leonine, has the cardio-protective action in H9c2 cells subjected with H/R.

SIRT1 overexpression or activation has protective actions on myocardial H/R injury by suppressing oxidative stress [28]. In addition, enhanced expression of SIRT1 mediated the protective effects on H/R injury in cardiomyocytes by various agents [29, 30]. We hypothesized that SIRT1 might also be a mediator in the protective effect of STA. The results demonstrate that STA increases the expression of SIRT1 in H9c2 cells with and without H/R insult. Furthermore, a specific SIRT1 inhibitor EX-527 could counteract the effects of STA on cell viability and caspase-3 activity in H9c2 cells with H/R insults. These results provide strong support for STA to attenuate H/R-induced damage in H9c2 cells by increasing SIRT1 expression. The regulation of SIRT1 by STA was also reported in high-glucose-induced endothelial cell senescence [31].

Our results showed that activation of Nrf2 might mediate the inhibition of oxidative stress by STA. Nrf2 is a crucial transcription factor induced by oxidative stress by upregulating the expression of antioxidant proteins. Under oxidative stress conditions, Nrf2 translocate from the cytoplasm into the nucleus and activates the transcription of many antioxidative genes, such as HO-1, SOD1, and SOD2. Nrf2 protein can reduce oxidative stress in various cardiovascular diseases [32]. Nrf2 protected myocardial H/R injury by reducing ROS production [33], including hypoxia-induced injury in cardiac H9c2 cells [34].This study provided new evidence that STA protected cardiomyocytes from H/R injury through activating Nrf2 since silencing Nrf2 attenuated the protection of STA against cell viability and caspase-3 activity in hypoxia/reoxygenation in H9c2 cells. To ascertain whether STA is a direct activator of the Nrf2 signal pathway, we investigated the relationship between SIRT1 and Nrf2. Our results also show the STA activated Nrf2/HO-1 signaling and EX-527, a SIRT1 inhibitor, reversed these effects. Conversely, enhanced SIRT1 protein by STA cannot be attenuated by transfection with Nrf2 siRNA. This indicates that the protective effect of STA is dependent on SIRT1 and Nrf2/HO-1 pathway. We, therefore, propose a SIRT1-Nrf2 pathway in myocardial H/R injury, which is supported by a previous report that the SIRT1-Nrf2 pathway mediates reduced apoptosis and oxidative stress in H/R-induced H9c2 cardiomyocytes [35].

The present study has some limitations, as follows. Firstly, we used in vitro cardiomyocyte H/R model to investigate the effects of STA. Therefore, there are potential differences between in vitro and in vivo H/R injury. The efficacy and molecular basis underlying the cardioprotection of STA should be further validated in vivo models of myocardial H/R injury. Secondly, we used the H9c2 cells, which is a specific cardiomyocyte line derived from the ventricular tissue of an embryonic rat and has the functions of both skeletal muscle and myocardium. So the physiological differences between H9c2 cells and primary adult rat cardiomyocytes cannot be overlooked. Thirdly, this study shows a potential SIRT1-Nrf2 pathway regulated by STA. How STA regulates upstream or downstream related- proteins of SIRT1, such as AMPK and NF-κB is unknown, and worth exploring.

## Conclusions

In conclusion, our study shows that STA is a new agent with cardioprotective effects against H/R injury. The related mechanisms might be associated with suppressed oxidative stress and apoptosis by SIRT1 and Nrf-2/HO-1 pathway. However, our study only evaluated the in vitro effect of STA, and its efficacy should be further validated in vivo models of myocardial H/R injury.

### Electronic supplementary material

Below is the link to the electronic supplementary material.

Supplementary Material 1

## Data Availability

All data generated or analyzed included in this article are available upon a reasonable request from the corresponding author.
