# Supplementary material for: Stachydrine ameliorates hypoxia reoxygenation injury of cardiomyocyte via enhancing SIRT1-Nrf2 pathway
Source: J Cardiothorac Surg. 2023 Sep 26;18:265. doi: 10.1186/s13019-023-02363-6 (PMC10521545; doi:10.1186/s13019-023-02363-6)
Supplement: Supplementary file 1 — Supplementary Material 1 [file 13019_2023_2363_MOESM1_ESM.ppt]

## Slide 1
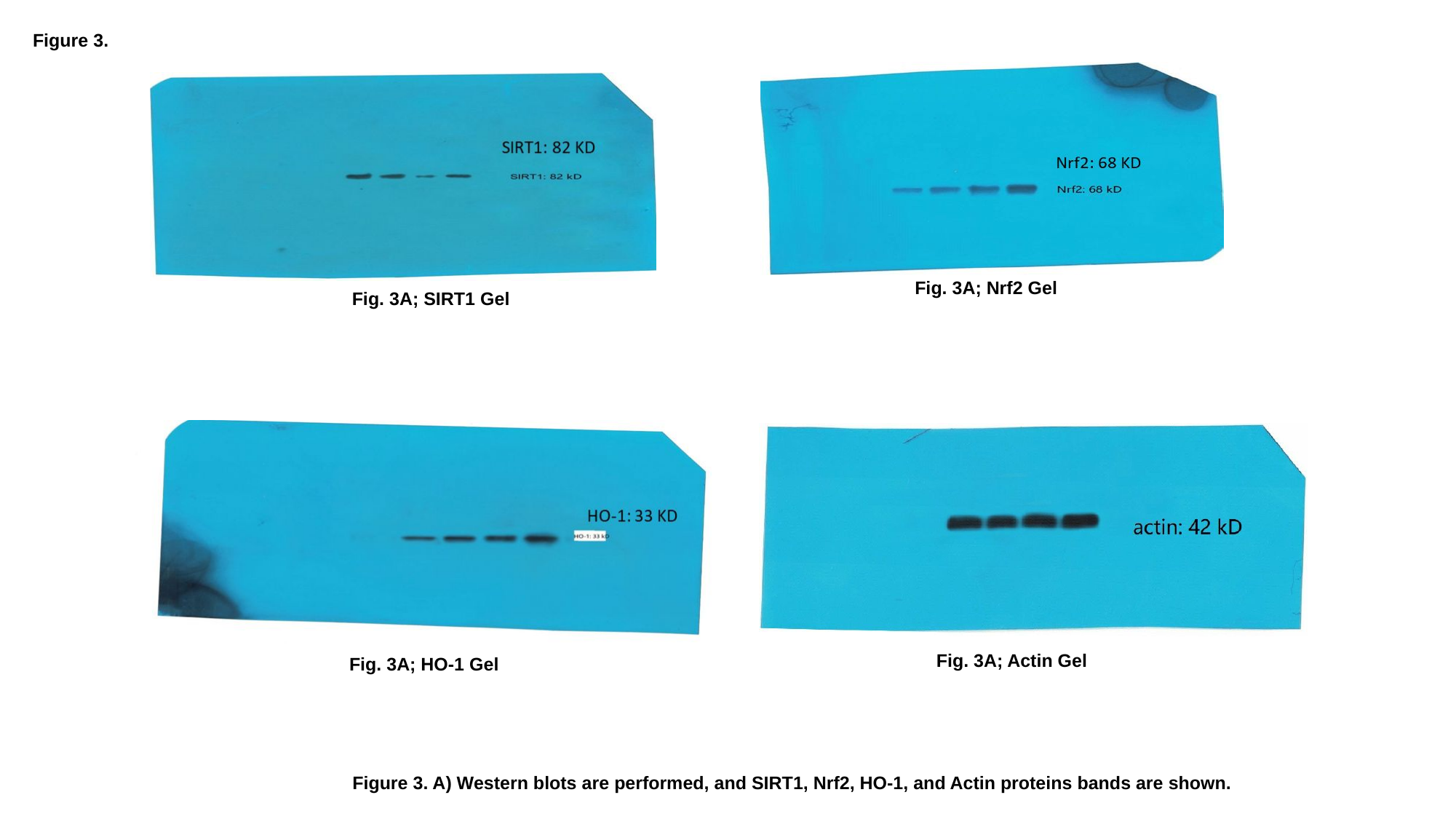

Figure 3.
Fig. 3A; Nrf2 Gel
Fig. 3A; SIRT1 Gel
Fig. 3A; Actin Gel
Fig. 3A; HO-1 Gel
Figure 3. A) Western blots are performed, and SIRT1, Nrf2, HO-1, and Actin proteins bands are shown.

## Slide 2
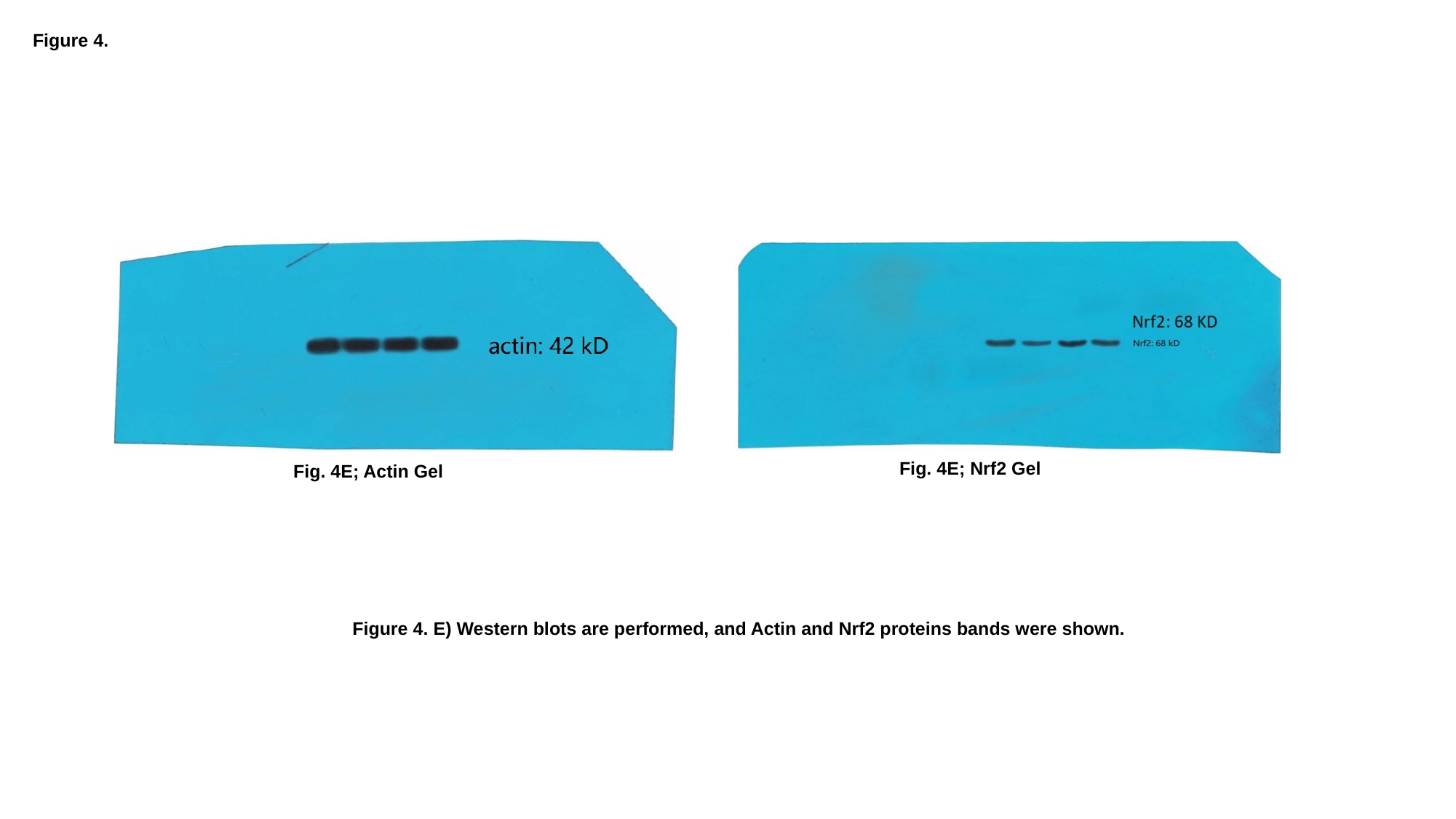

Figure 4.
Fig. 4E; Nrf2 Gel
Fig. 4E; Actin Gel
Figure 4. E) Western blots are performed, and Actin and Nrf2 proteins bands were shown.

## Slide 3
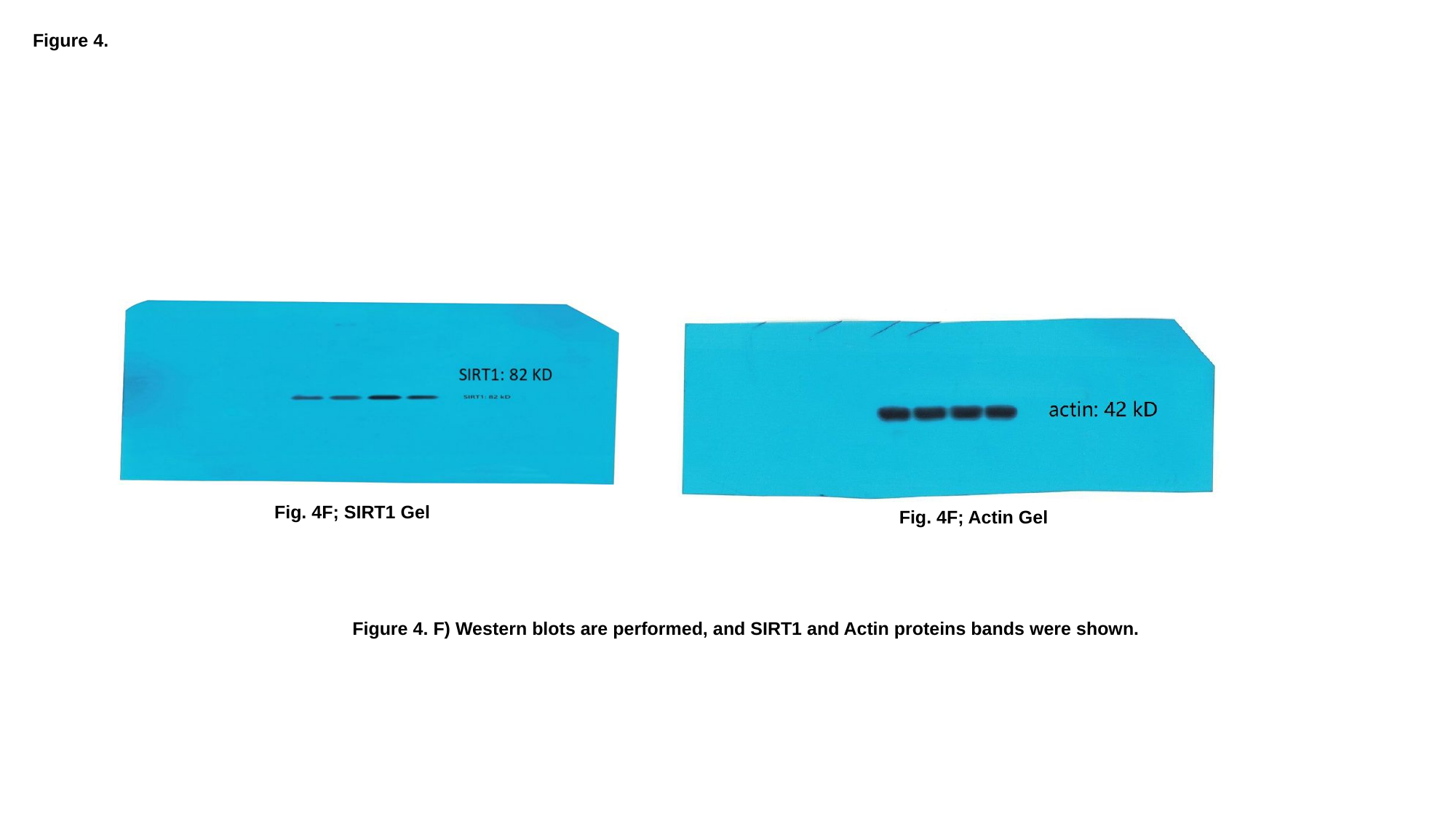

Figure 4.
Fig. 4F; SIRT1 Gel
Fig. 4F; Actin Gel
Figure 4. F) Western blots are performed, and SIRT1 and Actin proteins bands were shown.
